# Eosinophilic esophagitis-associated epithelial remodeling may limit esophageal carcinogenesis

**DOI:** 10.3389/falgy.2023.1086032

**Published:** 2023-03-29

**Authors:** Annie D. Fuller, Adam L. Karami, Mohammad Faujul Kabir, Alena Klochkova, Jazmyne L. Jackson, Anbin Mu, Yinfei Tan, Andres J. Klein-Szanto, Kelly A. Whelan

**Affiliations:** ^1^ Fels Cancer Institute for Personalized Medicine, Temple University Lewis Katz School of Medicine, Philadelphia, PA, United States; ^2^ Genomics Facility, Fox Chase Cancer Center, Philadelphia, PA, United States; ^3^ Histopathology Facility, Fox Chase Cancer Center, Philadelphia, PA, United States; ^4^ Department of Cancer & Cellular Biology, Temple University Lewis Katz School of Medicine, Philadelphia, PA, United States

**Keywords:** eosinophilic esophagitis, esophageal squamous cell carcinoma, esophageal epithelium, atopy, cell fate trajectory, cell differentiation

## Abstract

**Introduction:**

Under homeostatic conditions, esophageal epithelium displays a proliferation/differentiation gradient that is generated as proliferative basal cells give rise to suprabasal cells then terminally differentiated superficial cells. This proliferation/differentiation gradient is often perturbed in esophageal pathologies. Basal cell hyperplasia may occur in patients with gastroesophageal reflux disease (GERD), a condition in which acid from the stomach enters the esophagus, or eosinophilic esophagitis (EoE), an emerging form of food allergy. While GERD is a primary risk factor for esophageal cancer, epidemiological data suggests that EoE patients do not develop esophageal cancer.

**Methods:**

In order to investigate the impact of EoE and esophageal cancer specifically on the cellular landscape of esophageal epithelium, we perform single cell RNA-sequencing in murine models of EoE and esophageal cancer, specifically esophageal squamous cell carcinoma (ESCC). We further evaluate modules of co-expressed genes in EoE- and ESCC-enriched epithelial cell clusters. Finally, we pair EoE and ESCC murine models to examine the functional relationship between these pathologies.

**Results:**

In mice with either EoE or ESCC, we find expansion of cell populations as compared to normal esophageal epithelium. In mice with EoE, we detect distinct expansion of 4 suprabasal populations coupled with depletion of 2 basal populations. By contrast, mice with ESCC display unique expansion of 2 basal populations and 1 suprabasal population, as well as depletion of 2 suprabasal populations. Senescence, glucocorticoid receptor signaling, and granulocyte-macrophage colony-stimulating factor pathways are associated with EoE-enriched clusters while pathways associated with cell proliferation and metabolism are identified in ESCC-enriched clusters. Finally, our *in vivo* data demonstrate that exposure to EoE inflammation limits tumor burden of esophageal carcinogenesis.

**Discussion:**

Our findings provide the first functional investigation of the relationship between EoE and esophageal cancer and suggest that esophageal epithelial remodeling events occurring in response to EoE inflammation may limit esophageal carcinogenesis. This investigation may have future implications for leveraging allergic inflammation-associated alterations in epithelial biology to prevent and/or treat esophageal cancer.

## 1. Introduction

Stratified squamous epithelium of the esophagus exhibits an exquisite proliferation/differentiation gradient under homeostatic conditions. In esophageal epithelium, proliferation is confined to the basal cell layer (1, 2). As basal cells migrate outward toward the lumen, they execute a terminal differentiation program, giving rise to overlying suprabasal cells then terminally differentiated superficial cells (3). Maintenance of this proliferation/differentiation gradient, which is essential for epithelial barrier function, is perturbed in esophageal pathologies.

Basal cell hyperplasia is a feature associated with tissue remodeling in esophageal inflammation (i.e., esophagitis) and carcinogenesis. Esophageal squamous cell carcinoma (ESCC), the most common histological subtype of esophageal cancer worldwide, involves stepwise progression from basal cell hyperplasia to intraepithelial neoplasia, dysplasia, then frank carcinoma. The second predominate subtype of esophageal cancer, esophageal adenocarcinoma (EAC), is associated with gastroesophageal reflux disease (GERD) (4, 5), a condition in which acid-rich refluxate from the stomach enters the esophagus. Esophagitis may occur as a consequence of GERD, inducing both chemical injury and cytokine-mediated injury to esophageal mucosa as well as epithelial remodeling in the form of basal cell hyperplasia (6, 7). Esophageal epithelium of patients affected by the food allergen-mediated disease eosinophilic esophagitis (EoE) also often features basal cell hyperplasia in association with squamous differentiation and barrier defects (8, 9, 10, 11). While chronic inflammation resulting in epithelial remodeling is a hallmark of both GERD and EoE, epidemiological studies have failed to detect esophageal cancer in EoE patients (12, 13, 14).

Various studies have indicated negative associations between atopic disorders and cancer risk (15, 16, 17, 18, 19, 20, 21, 22, 23, 24). While emerging evidence supports immune-mediated mechanisms, including enhanced immunosurveillance and suppression of tumor-eradicating T helper (Th1) inflammation (25, 26, 27, 28), as potential factors supporting these epidemiological findings, we postulated that alterations in the cells that give rise to tumors (e.g., epithelial cells in the case of carcinoma) may also support impaired carcinogenesis in atopic individuals. To test this hypothesis, the current study explores the impact of ESCC and the food allergen-mediated disorder EoE upon the esophageal epithelial landscape using single cell RNA-Sequencing (scRNA-Seq). We focus on pairing EoE and ESCC for the current study as (1) robust murine models of these two conditions are available; and (2) ESCC arises from direct transformation of esophageal keratinocytes. Our studies reveal that exposure to EoE inflammation drives accumulation of unique suprabasal populations coupled with depletion of basal populations. By contrast, ESCC induces accumulation of basal populations. By contrast, ESCC induces accumulation of basal and suprabasal populations concomitant with depletion of suprabasal populations. Pathway analysis of genes displaying co-expression further indicates that epithelial remodeling in EoE is associated with senescence, glucocorticoid signaling, and granulocyte-macrophage colony-stimulating factor (GM-CSF) signaling while epithelial remodeling in ESCC is associated with cell proliferation and cell metabolism pathways. Finally, we report that that exposure to EoE inflammation limits ESCC carcinogenesis *in vivo*, providing the first functional interrogation of the relationship between these two esophageal pathologies.

## 2. Material and methods

### 2.1. Animal experiments

All research for the current study complies with all relevant ethical regulations. All murine studies were performed in accordance with a protocol approved by Temple University IACUC (Protocol Number: 5018). All animal experiments were conducted in accordance with institutional guidelines for animal research. All mice were maintained under controlled conditions with a 12 h light/dark cycle at an appropriate temperature and humidity. C57BL/6 mice (Cat# 000664) were obtained from the Jackson Laboratory (USA) and bred for experiments. In mice, administration of the food allergen ovalbumin (OVA; A5503, Sigma-Aldrich, St. Louis, MO, USA) coupled with cutaneous challenge with the Vitamin D analog MC903 (calcipotriol; 2700, Tocris, Bristol, UK) promotes esophageal eosinophilic infiltrates and food impactions (22, 29) and oral administration of the carcinogen 4-nitroquinoline 1-oxide (4NQO; N8141, Sigma-Aldrich) induces esophageal tumors that recapitulate histological and molecular features of human ESCC (30, 31).

The following procedures were conducted for individual induction of EoE and ESCC. EoE-like inflammation was induced using the previously described MC903/OVA mouse model (29, 32) over a period of 32 days. For 12 days, ears of mice were scraped with a scalpel blade then 20 *μ*l MC903 (10 μM dissolved in 100% ethanol) was applied to each ear followed by 10 *μ*l OVA (10 mg/mL in PBS). From days 15–32, mice were subjected to oral gavage with 100 *μ*l OVA (500 mg/mL in water) every other day and provided *ad libitum* access to drinking water supplemented with OVA (15 g/L). Mice were euthanized at day 32 and esophagi were dissected for scRNA-Seq analysis. To induce ESCC, mice were administered 4NQO (100 µg/ml in 2% propylene glycol) for 16 weeks *via* drinking water. 4NQO was then withdrawn for a period of 8 weeks. At the end of this 24-week protocol, mice were euthanized, and esophagi were dissected for scRNA-Seq analysis. Untreated wild type C57BL/6 mice served as controls.

For experiments combining EoE and ESCC, C57BL/6 mice were randomly assigned to one of 4 treatment groups and the following procedures were conducted over a period of 28 weeks.

 • **Mice in Group 1 [EoE (-) ESCC (-)]** served as a MC903-only control. As MC903 induces dermatitis (33), it is important to control for the effects of this agent with regard to esophageal carcinogenesis. Mice in group 1 were treated with MC903 for 12 days and vehicle control for ovalbumin (OVA) throughout the 32-day experiment. For 12 days, ears of mice were scraped with a scalpel blade then 20 *μ*l MC903 (10 *μ*M dissolved in 100% ethanol) was applied to each ear followed by 10 *μ*l PBS. From days 15–32, mice were subjected to oral gavage with 100 *μ*l water every other day. At day 33, mice were provided drinking water with 2% propylene glycol for 16 weeks. Mice were then administered normal drinking water for 8 weeks. For the final 24 weeks of the experiment, mice were subjected to oral gavage with water every other day for the last week of each month.
 • **Mice in Group 2 [EoE (+) ESCC (-)]** served to assess the long-term effects of EoE exposure with regard to esophageal epithelial alterations. Mice in group 2 were treated with MC903 and OVA for a period of 32 days. For 12 days, ears of mice were scraped with a scalpel blade then 20 *μ*l MC903 (10 *μ*M dissolved in 100% ethanol) was applied to each ear followed by 10 *μ*l OVA (10 mg/ml in PBS). From days 15–32, mice were subjected to oral gavage with 100 *μ*l OVA (500 mg/ml in water) every other day and provided *ad libitum* access to drinking water supplemented with OVA (15 g/L). At day 33, mice were provided drinking water with 2% propylene glycol for 16 weeks. Mice were then administered normal drinking water for 8 weeks. During the 24-week period following EoE induction, mice were subjected to oral gavage with 100 *μ*l OVA (500 mg/ml in water) every other day and provided *ad libitum* access to drinking water supplemented with OVA (15 g/L) for the last week of each month.
 • **Mice in group 3 [EoE (-) ESCC (+)]** served to assess the effect of 4NQO alone on esophageal carcinogenesis. Mice in group 3 were treated with MC903 only for a period of 32 days. For 12 days, ears of mice were scraped with a scalpel blade then 20 *μ*l MC903 (10 *μ*M dissolved in 100% ethanol) was applied to each ear followed by 10 *μ*l PBS. From days 15–32, mice were subjected to oral gavage with 100 *μ*l water every other day. At day 33, mice were provided drinking water with 4NQO (100 µg/mL in 2% propylene glycol) for 16 weeks. Mice were then administered normal drinking water for 8 weeks. For the final 24 weeks of the experiment mice, were subjected to oral gavage with water every other day for the last week of each month.
 • **Mice in Group 4 [EoE (+) ESCC (+)]** served to assess the effects of EoE exposure on esophageal carcinogenesis. Mice in group 4 were treated with MC903 and OVA for a period of 32 days. For 12 days, ears of mice were scraped with a scalpel blade then 20 *μ*l MC903 (10 *μ*M dissolved in 100% ethanol) was applied to each ear followed by 10 *μ*l OVA (10 mg/ml in PBS). From days 15–32, mice were subjected to oral gavage with 100 *μ*l OVA (500 mg/ml in water) every other day and provided *ad libitum* access to drinking water supplemented with OVA (15 g/L). At day 33, mice were provided drinking water with 4NQO (100 µg/ml in 2% propylene glycol) for 16 weeks. Mice were then administered normal drinking water for 8 weeks. During the 24-week period following EoE induction, mice were subjected to oral gavage with 100 *μ*l OVA (500 mg/ml in water) every other day and provided *ad libitum* access to drinking water supplemented with OVA (15 g/L) for the last week of each month.

### 2.2. Esophageal processing

Whole esophagi were dissected from each mouse and opened longitudinally. Images were captured using an Olympus MVX10 microscope. Tumor number was counted and tumor area (mm^2^) was calculated using ImageJ. Tumor load was calculated as tumor number x tumor area for each mouse. For histological analyses, esophagi were fixed with 10% neutral buffered formalin (5701; Epredia; Kalamazoo, MI, USA) for 12 h at 4°C. Tissues were washed with PBS then stored in 70% ethanol at 4°C prior to paraffin embedding. Hematoxylin and eosin (H&E) staining was performed at Fox Chase Cancer Center Histopathology Facility (Philadelphia, USA). H&E-stained slides were evaluated for presence of intraepithelial neoplasia (IEN), early ESCC, and invasive ESCC and percent of epithelium occupied by any of these lesions was calculated. Slides were imaged using Leica DM1000 LED microscope (474301; Leica; Wetzlar, Germany). To generate a single cell suspension, esophageal epithelium-enriched tissue layer was peeled from muscle then incubated in 1 ml 1X Dispase I (354235; Corning; Bedford, MA, USA) diluted 1:4 in Hank's Balanced Salt Solution (HBSS; 14025-076; Gibco; Grand Island, NY, USA) for 10 min at 37°C with shaking at 1,000 RPM (5384; Eppendorf F1.5 Thermomixer; Hamburg, Germany). Following removal from Dispase I, esophageal epithelium was minced with sharp scissors then incubated in 1 ml of 0.25% Trypsin-EDTA (25-053-CI; Corning; Manassas, VA, USA) for 10 min at 37°C with shaking at 1,000 RPM. Trypsin and tissue pieces were forced through 70 *μ*m cell strainer into 50 ml conical tube containing 4 ml soybean trypsin inhibitor (STI; 9035-81-8; Grand Island, NY, USA). Cells were pelleted at 1,300 RPM for 3 min then resuspended in 500 *μ*l complete mouse keratinocyte–serum-free medium (37010022; Gibco; Carlsbad, CA, USA). Cell number and viability were measured using Automated Cell Count (AMQAX1000, Invitrogen Countess II, Bothell, WA, USA) by mixing 5 *μ*l cell suspension with 5 *μ*l 0.4% trypan blue solution (T10282; Invitrogen; Eugene, OR, USA).

### 2.3. scRNA library preparation and sequencing

Single cell droplets were generated with inDrop according to manufacturer's protocols. 5,000–7,000 cells were collected to make cDNA at the single cell level. Full-length cDNA with Unique Molecular Identifiers (UMI) was synthesized *via* reverse-transcription in the droplet. After PCR amplification and purification, cDNA was fragmented to ∼270 bp and the Illumina adapters with index were ligated to fragmented cDNA.

### 2.4. Deconvolution of scRNA-Seq reads

FASTQ files from the sequencing run were downloaded from Illumina's BaseSpace sequence hub. To resolve the mapping of cellular barcodes and Unique Molecular Identifiers (UMIs), UMI-tools (v1.0.0) was used to whitelist and extract the barcodes. Likely barcodes were found using the whitelist function of UMI-tools (34), which searches for the inDrop regular expression pattern of “(?P < cell_1 > .(7))(?P < discard_1 > GAGTGATTGCTTGTGACGCCTT){s<=2}(?P < cell_2 > .{8})(?P < umi_1 > .{6})T(42).*” in the R2 of the read pairs. Using a whitelist of likely barcodes, the extract function relocated both the cell barcode and the unique molecular identifier found in the same read to the read name in the FASTQ files. The extraction retains the information of unique cellular barcodes while enabling correct read mapping of genes without the attached cell and transcript identifiers. Reads were then mapped using the aligner STAR (v2.7.3). First, the murine genome index was made using the M23 GRCm38 genomic sequence from GENCODE. Using the index, all read pairs were aligned to the genome for an output of BAM files. To limit the variance of amplification on the length of transcripts, the UMI-tools dedup tool was used to deduplicate the reads, which deduplicates UMIs that match to a single transcript. The resulting deduplicated alignments were then mapped to the murine gene transfer file (GTF) from GENCODE using featureCounts to count the number of reads mapping to each gene in the genome. Finally, the UMI-Tools count function was used to summarize the gene counts in each cell in each sample to give an output of a matrix with gene names and cell barcodes.

### 2.5. Data filtering and initial clustering, and exclusion of non-epithelial cells

The matrices for each sample were imported and transformed into Seurat (v3.2.2) objects for further processing. Genes expressed in 3 or fewer cells were excluded from analysis. To remove doublets and dead cells, the distribution of unique gene count per cell as well as total RNA counts were assessed. Cells with unique gene counts under 250 and over 3,500, as well as total RNA counts under 100 and over 9,500 were excluded. To equalize cell counts between conditions, 19,041 total cells sequenced were downsampled to 1,500 cells per condition (4,500 cells total; approximately the number of cells from EoE samples).

Using Monocle3 on R (v1.0.0), a gene expression matrix (GEM) was generated from Seurat objects and used to create a cell data set (CDS) object. Batch effects were effectively removed by the regression function in Monocle3. The resulting dataset was then scaled and centered for dimensionality reduction. Principal component analysis (PCA) was used for initial dimensionality reduction and later for clustering, projecting each cell on the top 50 principal components according to the default specifications in Monocle3. The PCA principal components were then used as input to the Uniform Manifold Approximation and Projection (UMAP) dimensionality reduction procedure for visualization, embedding into 2 components for visualization. Cell population clusters were developed using Monocle3. 13 distinct cellular populations were found at resolution 3 × 10^−3^, each with a shared transcriptomic profile across different samples.

### 2.6. Gene module discovery

To determine transcriptomic programs prevalent in the different subsections of the epithelial dataset and across pathological conditions, we used Monocle3's gene module functionality. Briefly, Monocle3 performs UMAP on genes with cells as features to find distinct groups of genes locally co-expressed in the spatio-pseudotemporal trajectory inferred within the cellular UMAP space. 20 such gene modules were found. Modules of pathological interest were imported into QIAGEN Ingenuity Pathway Analysis (IPA) for core analysis.

### 2.7. Cell cycle phase prediction and pseudotemporal trajectory inference

To instantiate pseudotemporal trajectory inference that simulates the biology of the esophageal epithelium, we first determined the cell cycle phases of the dataset's cells. These phases were determined by Seurat's CellCycleScoring function, which uses markers of S and G2/M phases to approximate the cell cycle phase of each cell. With the phases calculated, we chose the root node as the crux of the G1/G0 and G2M/S. Calculation of the pseudotime values then chose the root point as the start of the trajectory, subsequently inferring the cellular lineages radiating from the root.

### 2.8. Histological analysis

Whole esophagi were dissected and fixed with 4% paraformaldehyde (#J19943-K2; Thermo Scientific, Waltham, MA, USA) or 10% neutral buffered formalin (5701; Epredia, Kalamazoo, MI, USA) for 12 h at 4°C. Tissues were washed with PBS then stored in 70% ethanol at 4°C prior to paraffin embedding. Slides from untreated controls (*n* = 5), mice with MC903/OVA-induced EoE (*N* = 5) and 4NQO-induced ESCC (*n* = 5) were stained with anti-p21 [EPR18021] (Ab188224; Abcam, Waltham, Boston, MA, USA) at 1:1,000 and counterstained with hematoxylin using previously described methods (32). Slides were imaged using Leica DM 1000 LED microscope (Leica, Germany).

### 2.9. Statistical analysis

Unpaired Student's *t*-test, two-way ANOVA followed by Tukey's multiple comparisons, Wilcoxon signed-rank test, and Fisher's exact test (as indicated in figure legends) were used for statistical evaluation of data. *p* < 0.05 was used as the threshold for statistical significance. Statistical analysis was performed using GraphPad Prism (GraphPad Software, La Jolla, CA, USA).

## 3. Results

### 3.1. Identification and characterization of esophageal epithelial cell populations along the basal/superficial cell axis in mice with EoE or ESCC

**Figure 1:**
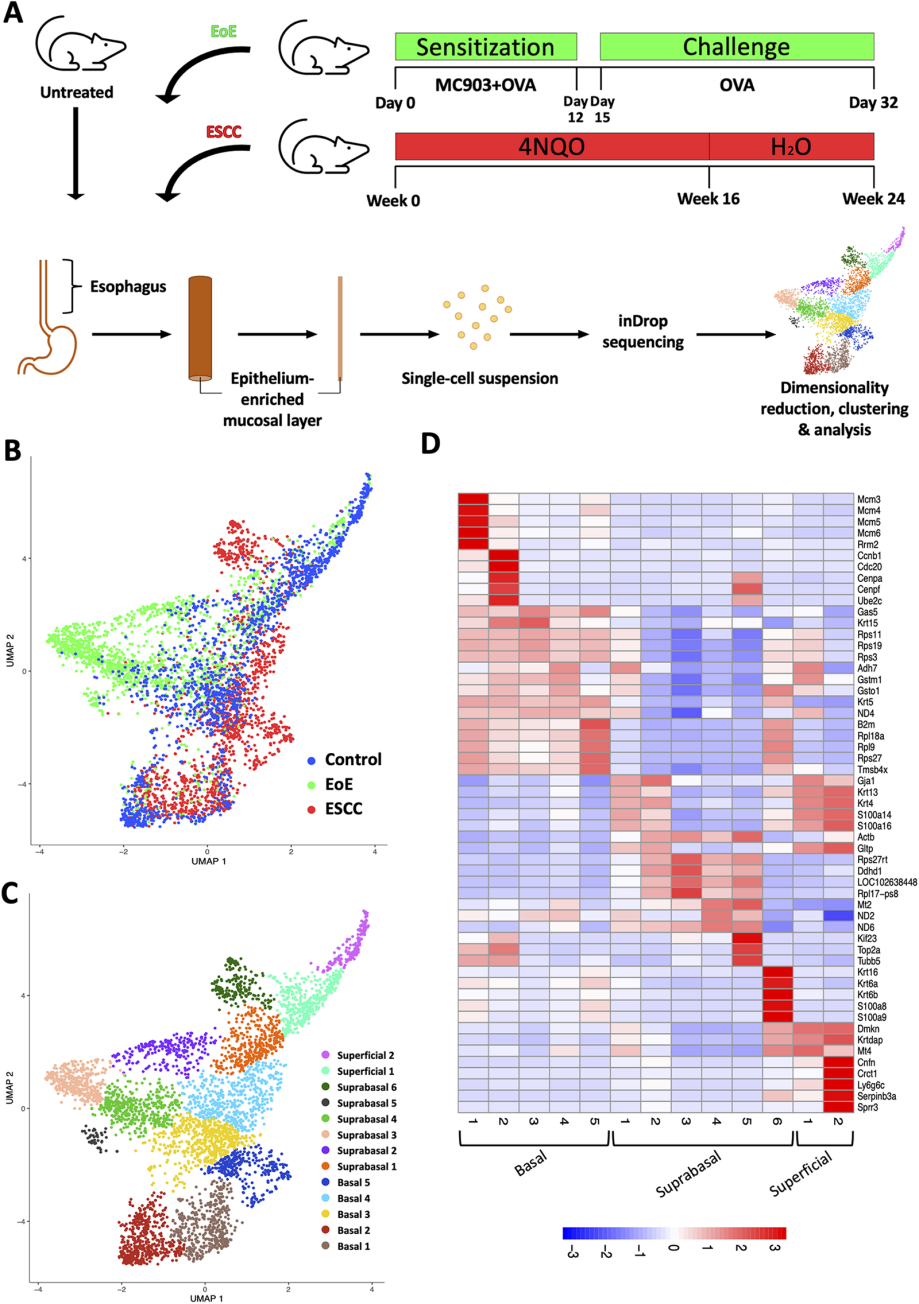
Single cell profiling of esophageal epithelium from mice treated with eosinophilic esophagitis (EoE) or esophageal squamous cell carcinoma (ESCC). (**A**) Schematic of experimental approach. MC903 and Ovalbumin (OVA) were used to induce EoE over 32 days in C57B6 mice (*n* = 4). During the 12-day sensitization period, mice were treated epicutaneously with MC903 and OVA. Challenge with OVA in drinking water and *via* gavage (3x/week) was conducted from day 15 through day 32. ESCC was induced in C57B6 mice (*n* = 3) using the chemical carcinogen 4-nitroquinoline-1-oxide (4NQO). 4NQO was administered *via* drinking water for 16 weeks followed by an 8 week wash out period. Untreated C57B6 mice (*n* = 6) served as controls. In each mouse, esophageal epithelium-enriched mucosal layer was peeled from underlying muscle then enzymatically digested to generate a single cell suspension that was subjected to single cell RNA-Sequencing using the inDrop platform. (**B,C**) Uniform Manifold Approximation and Projection plot (UMAP) shows distribution of all cells from the single cell RNA-Sequencing dataset by condition with blue identifying cells from control mice, green identifying cells from mice with EoE, and red identifying cells from mice with ESCC in (**B**) or by distinct cell populations identified using the R-based program Monocle3 in (**C**). (**D**) Heat map shows z-score scaled normalized expression of up to 5 of the most uniquely upregulated genes in each population across all clusters. Red represents upregulation and blue represents downregulation.

**Figure 2:**
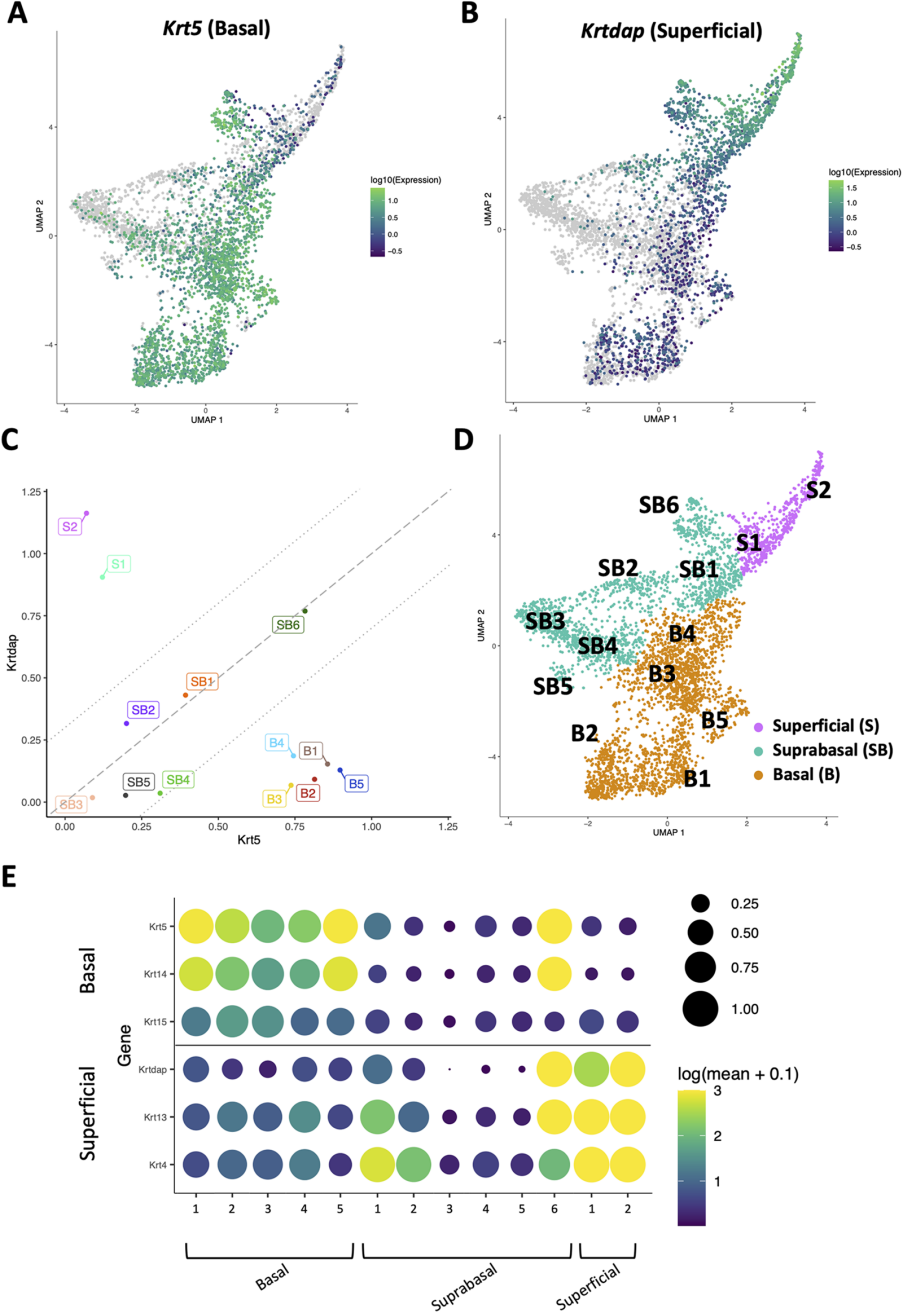
Identification of basal, suprabasal, and superficial cells across dataset. (**A,B**) Normalized log_10_ expression gradients of Keratin 5 (*Krt5*), an established marker of basal esophageal epithelial cells in A, or Keratinocyte Differentiation-Associated Protein (*Krtdap*), an established marker of differentiated esophageal epithelial cells in B are shown across the single cell RNA-Sequencing dataset. Green indicates enrichment while purple indicates inhibition. (**C**) Normalized log_10_ expression of *Krt5* vs. normalized log_10_ expression of *Krtdap* expression was plotted for all populations identified in the single cell RNA-Sequencing dataset. Populations falling within the strip defined by lines with a slope of 1 passing through points *(0, −0.3)* and *(0, 0.3)* were define as suprabasal (SB). Populations falling below this strip were defined as basal (**B**) and those above this strip were identified as superficial (**S**). (**D**) Basal (B; denoted in orange), suprabasal (SB; denoted in teal), and superficial (S; denoted in pink) clusters identified on a Uniform Manifold Approximation and Projection plot (UMAP) of the entire single cell RNA-Sequencing dataset. (**E**) Cluster-average expression z-scores of putative basal and differentiated markers are shown for each population in the single cell RNA-Sequencing dataset. Circle size reflects percentage of cells with non-zero expression level for indicated genes. Color intensity reflects average expression level across all cells within each cluster with yellow indicating enrichment and purple indicating inhibition.

To define how EoE and ESCC influence the cellular landscape of esophageal epithelium, we performed scRNA-Seq on mice treated with MC903/OVA to induce EoE (*n* = 3), 4NQO to induce ESCC (*n* = 4), or untreated controls (*n* = 6). Epithelium was peeled from dissected esophagi of all mice and subjected to scRNA-Seq using the inDrop platform (Figure 1A). The resulting dataset consisted of 4,500 cells across the three groups that were then subjected to unsupervised dimensionality reduction and visualized by UMAP (Figure 1B). Monocle3-based clustering within the dataset further revealed 13 epithelial cell populations and up to 5 of the most uniquely upregulated genes in each population (Figures 1C,D). To establish the identity of these populations, we employed *Krt5* (encoding Cytokeratin 5) and *Krtdap* (encoding Keratin Differentiation-Associated Protein) as these are respective markers of basal and superficial cells (Figures 2A,B) (35). Relative *Krt5* vs. *Krtdap* expression for all populations was then plotted and evaluated relative to a linear slope of 1 through the origin point. Two groups of populations were visually distinguished by their distance from this line by way of high expression of one marker and low expression of the other. To distinguish these groups, a strip was generated which created a range of points that fall within a vertical distance of 0.3 from the origin line. Populations that fall within this strip were defined as suprabasal, as their expression of either marker does not sufficiently dominate the other. Among populations that fall outside the strip, those with predominant expression of *Krt5* were defined as basal and those with predominant expression of *Krtdap* were defined as superficial (Figure 2C). Using these methods, 5 basal, 6 suprabasal, and 2 superficial populations were identified within the dataset (Figures 2C,D). Mapping of additional established markers of basal and superficial cells in esophageal epithelium supported these classifications (Figure 2E).

### 3.2. Effects of EoE and ESCC on cell populations and trajectories in esophageal epithelium

**Figure 3:**
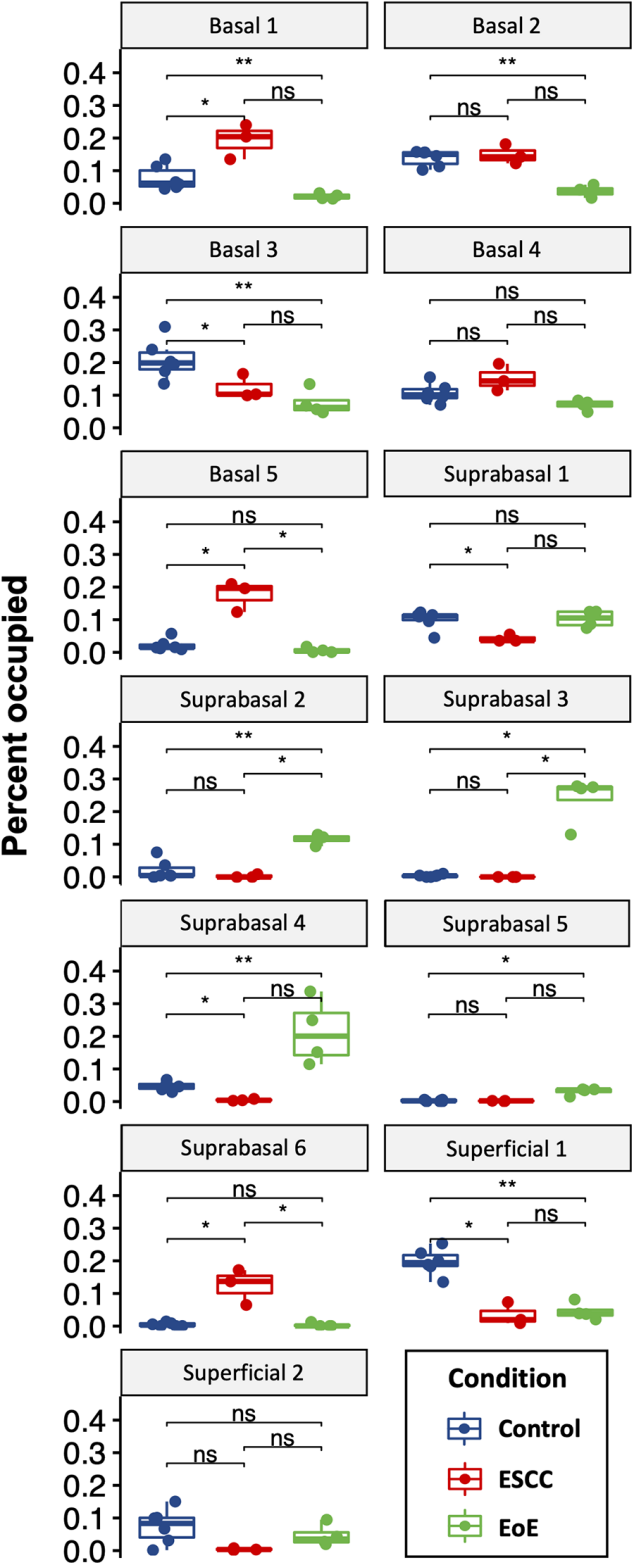
Effects of eosinophilic esophagitis (EoE) and esophageal squamous cell carcinoma (ESCC) on representation of esophageal epithelial cell populations. Proportion of each cell as a fraction of all cells in the single cell RNA-Sequencing dataset with blue indicating control mice, red indicating mice with ESCC, and green indicating mice with EoE. Each individual scatter point represents proportion indicated per biological replicate, box indicates quartiles, whiskers indicate minima and maxima. Mean is indicated by line striking through box. *, *p* < 0.05; **, *p* < 0.01; ***, *p* < 0.001 by Wilcoxon signed-ranked test with adjustment for multiple comparisons.

**Figure 4:**
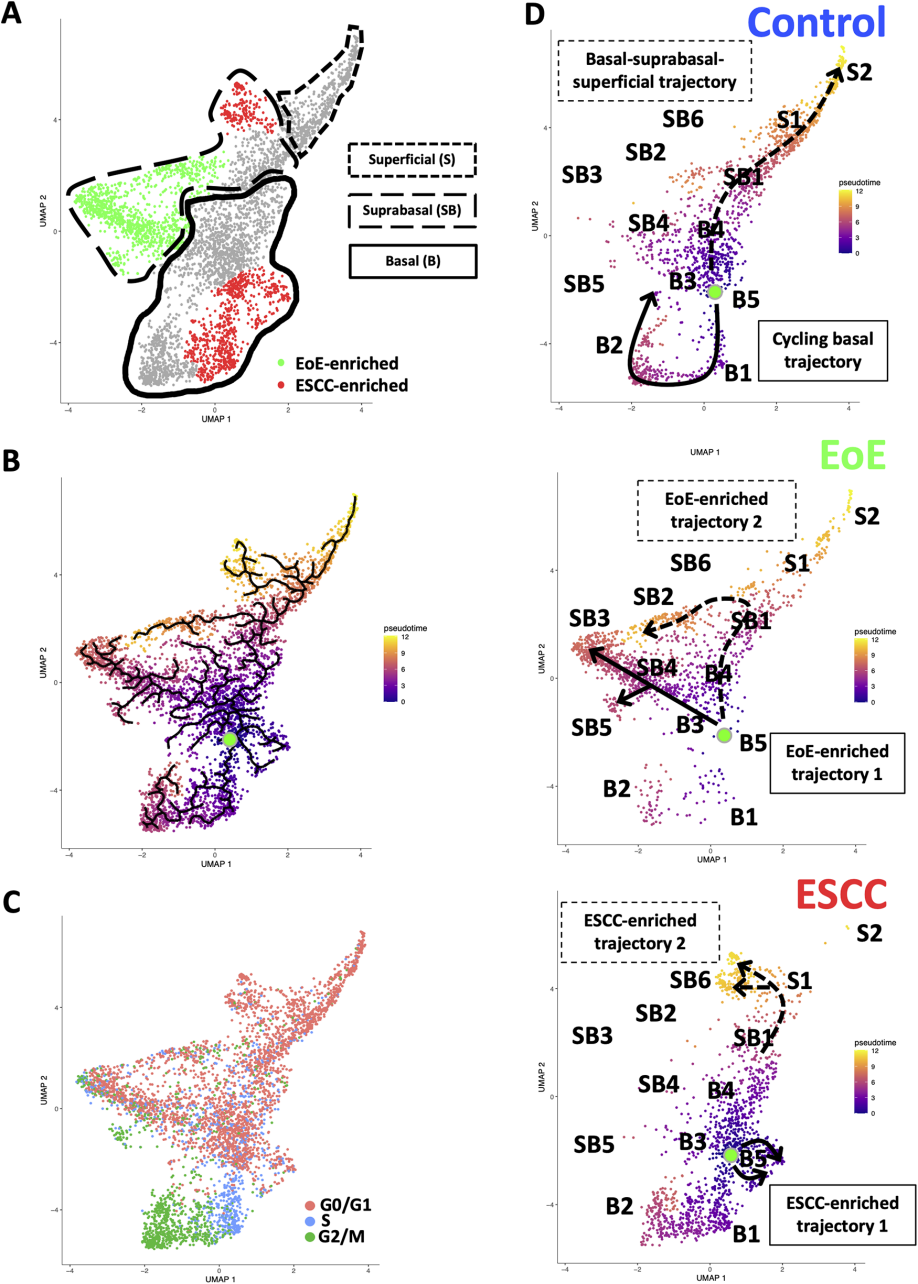
Effects of eosinophilic esophagitis (EoE) and esophageal squamous cell carcinoma (ESCC) on esophageal epithelial cell fate. (**A**) Uniform Manifold Approximation and Projection plot (UMAP) of entire single cell RNA-Sequencing dataset with cell populations found to be significantly enriched in mice with EoE or mice with ESCC colored green or red, respectively. Basal cells are enclosed in solid lines, suprabasal cells are enclosed in dashed lines, and superficial cells are enclosed in lines made of square dots. (**B**) Monocle3 UMAP visualization of all cells in single cell RNA-Sequencing dataset. Each cell is colored by its inferred pseudotime value with dark purple representing the earliest cells and bright yellow representing the latest cells in the trajectory. Green dot indicates supervised pseudotime root. Black lines represent putative cell fate trajectories. (**C**) Expression of genes associated with each phase of the cell cycle were labeled on UMAP. (**D**) Pseudotime projections of cells from untreated controls or mice with EoE or ESCC. Lines indicate predicted trajectories with trajectory names noted. Identification numbers show the location of individual basal (**B**), suprabasal (SB), and superficial (**S**) cell populations in the pseudotime projections.

Evaluation of our dataset separated by treatment group revealed that mice with EoE and ESCC both displayed expansion and depletion of cell populations (Figure 3, Supplementary Figure S1). Expansion of populations suprabasal 2, 3, 4, and 5 and depletion of populations basal 1 and 2 was unique to mice with EoE. Expansion of populations basal 1, 5, and suprabasal 6 and depletion of populations suprabasal 1 and 4 was unique to mice with ESCC (Figure 3). In mice with EoE, enriched cell populations were exclusively suprabasal (Figure 4A) and depleted populations were exclusively basal. By contrast, mice with ESCC exhibited expansion of both suprabasal and basal cells (Figure 4A) and depleted populations were exclusively suprabasal (Figure 4A).

To investigate how EoE and ESCC influence cell fate in esophageal epithelial cells, we continued to perform pseudotime analysis (Figure 4B) coupled with evaluation of cell cycle-associated gene expression (Figure 4C). Based upon our prior characterization of the cellular landscape of esophageal epithelium (35), the pseudotime root was set at the G0/G1-enriched basal population immediately preceding the S phase-enriched basal population. Through visual analysis of the pseudotime projection, we detected two predominant trajectories in control mice: a cycling basal cell trajectory and a basal-suprabasal-superficial trajectory (Figure 4D), consistent with our previous studies (35). Pseudotime analysis further suggests that EoE-enriched suprabasal cells arise from terminal trajectories that are present in esophageal epithelium of control animals, but to a limited extent (Figure 4D). EoE-enriched trajectory 1 branches directly from basal cells to give rise to EoE-enriched populations suprabasal 3, 4, and 5, while EoE-enriched trajectory 2 branches from the suprabasal pool to give rise to EoE-enriched population suprabasal 2 (Figure 4D). Population basal 1 is prevalent in the basal-suprabasal trajectory in control mice and is further expanded in mice with ESCC (Figure 4D). By contrast, populations basal 5 and suprabasal 6 are two terminal cell fates that are minimally represented in control mice but are highly enriched in mice with ESCC, representing ESCC-enriched trajectories 1 and 2 (Figure 4D). In ESCC-enriched trajectory 1, population basal 5 branches directly from basal cells found in the normal epithelium (Figure 4D). In ESCC-enriched trajectory 2, population suprabasal 6 branches off from the suprabasal cell pool common to mice in all experimental groups (Figure 4D), representing an aberration from the normal basal-suprabasal-superficial trajectory in esophageal epithelium.

**Figure 5:**
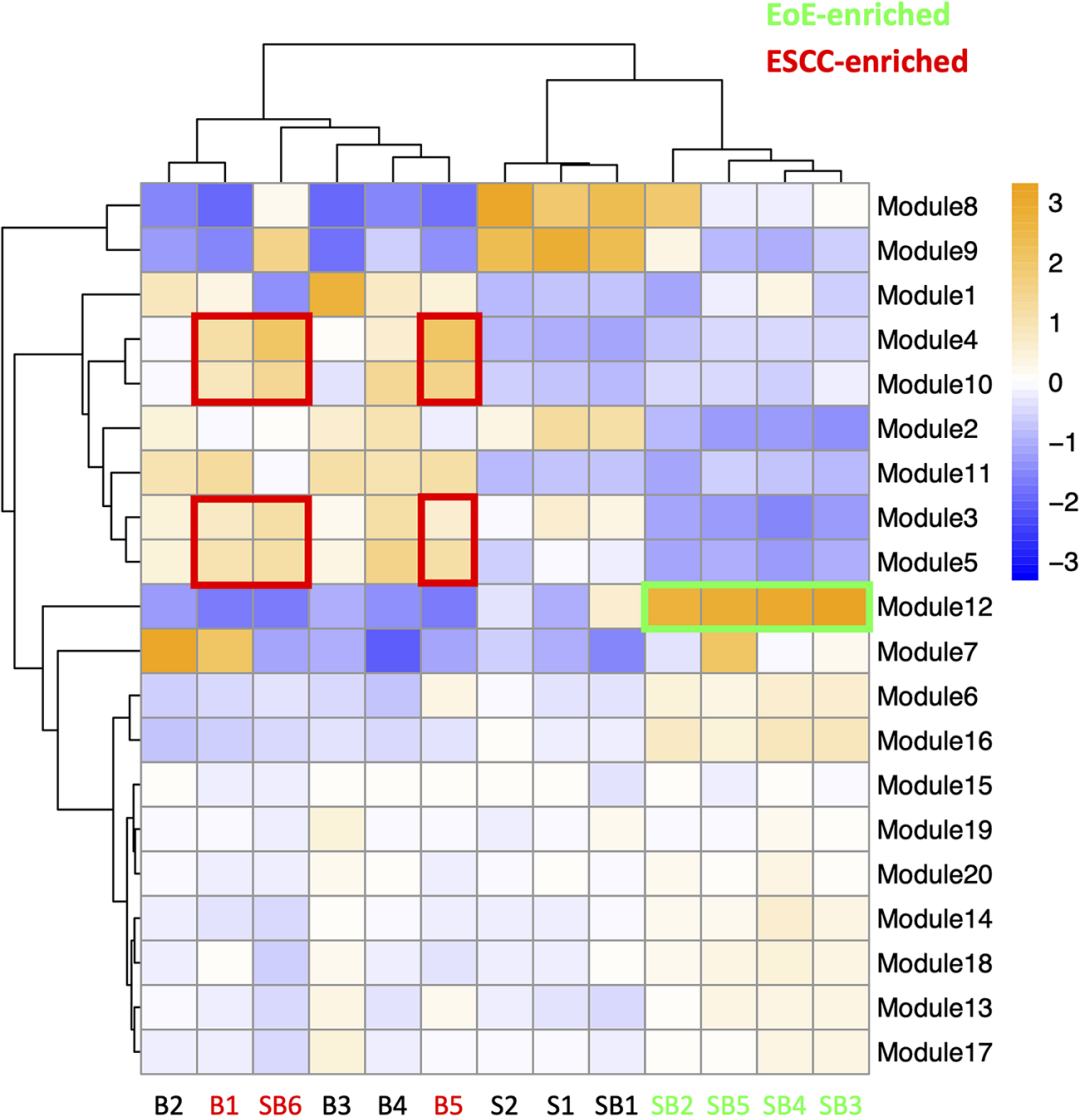
Modules of co-expressed genes and their z-score scaled normalized expression in cell populations across the single cell RNA-sequencing dataset. A positive value (orange) indicates upregulation while a negative value (blue) indicates inhibition of the respective genes in each module. Cell populations and modules that are enriched in mice with eosinophilic esophagitis (EoE) are highlighted in green. Cell populations and modules that are enriched in mice with esophageal squamous cell carcinoma (ESCC) are highlighted in red.

**Figure 6:**
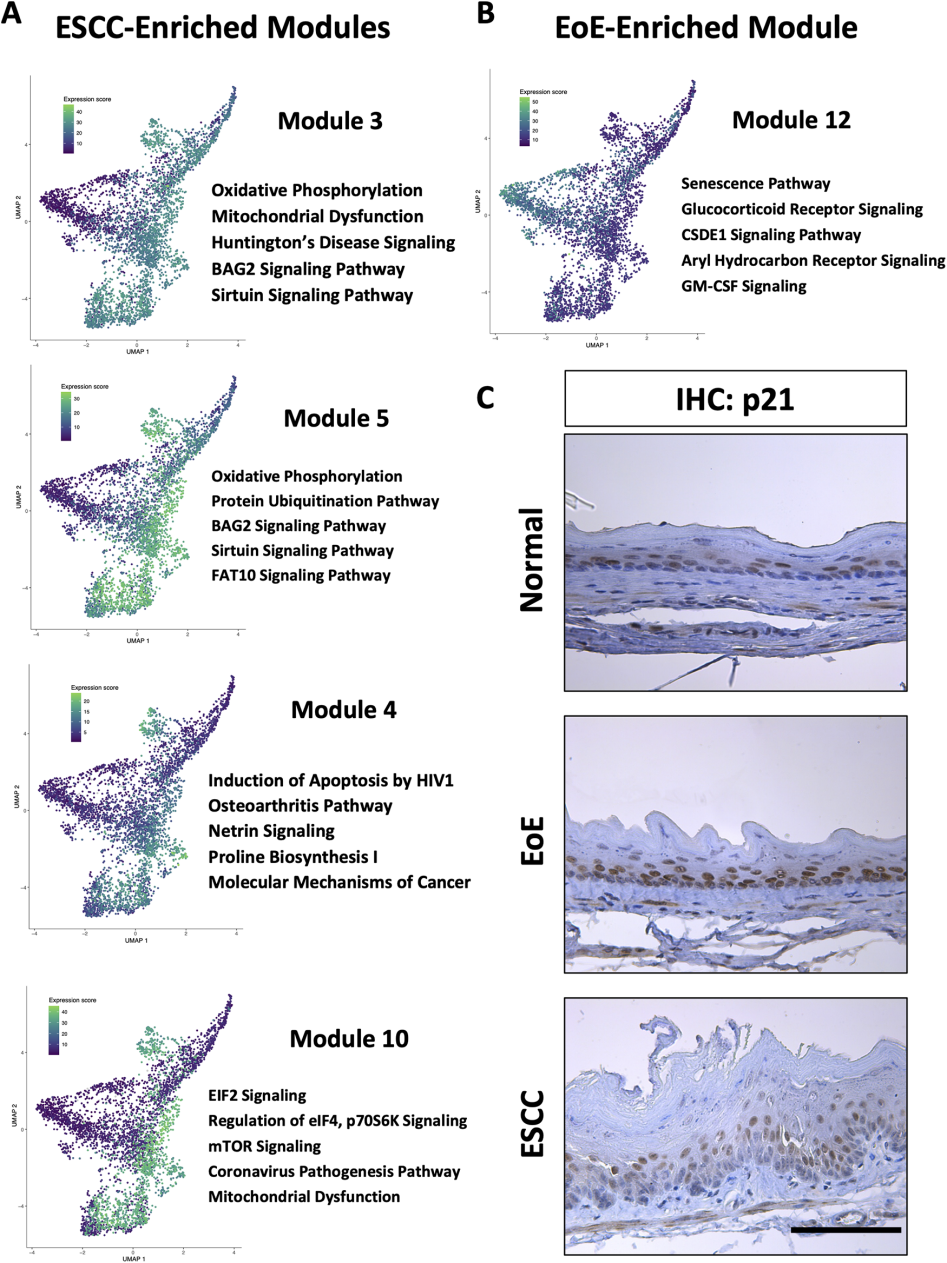
Pathways associated with cell populations that are enriched in mice with esophageal squamous cell carcinoma (ESCC) or eosinophilic esophagitis (EoE). (**A,B**) Ingenuity Pathway Analysis (IPA) identified cellular processes predicted to be significantly altered in co-expressed genes in modules that are associated with populations enriched in mice with ESCC in A or EoE in B. Associated Uniform Manifold Approximation and Projection plots (UMAPs) show normalized expression levels of each module across the entire single cell RNA-Sequencing dataset with green indicating high expression and purple indicating low expression. Top 5 pathways predicted to be most significantly associated with each gene module are listed. (**C**) Immunohistochemistry for p21 in mice with MC903/Ovalbumin-induced EoE, 4-nitroquinoline 1-oxide-induced ESCC or untreated controls (designated normal). Scale bar, 100 *μ*m.

To investigate the molecular features associated with cell populations and trajectories that are enriched in mice with EoE and ESCC, co-expressed genes were grouped into modules that were differentially expressed between populations (Figure 5). Gene modules 3, 4, 5, and 10 were predicted to be enriched in all ESCC-specific clusters while module 12 was predicted to be highly enriched in all EoE-specific clusters (Figure 5). Pathway analysis revealed that genes in ESCC-enriched modules were associated with proliferation, such as EIF2 signaling, regulation of EIF4 and p70S6 kinase, and mTOR signaling in module 10 (Figure 6A; Table 1). Other pathways regulated in ESCC-enriched populations were involved in altered metabolism, such as mitochondrial dysfunction and oxidative phosphorylation in modules 3, 5 and 10 (Figure 6A; Table 1). Additionally, genes in module 4 were associated with molecular mechanisms of cancer (Figure 6A; Table 1). Whereas genes associated with proliferation were linked to ESCC-enriched clusters, EoE-enriched clusters were associated with the senescence pathway (Figure 6B; Table 1). Immunohistochemistry for the senescence-associated cell cycle inhibitor p21 revealed increased staining intensity in esophageal epithelium of mice with EoE as compared to controls and mice with ESCC (Figure 6C). Additionally, while p21 was almost exclusively localized to suprabasal cells in normal controls and mice with ESCC, expression of p21 in basal cells was apparent in mice with EoE (Figure 6C). Genes associated with glucocorticoid receptor signaling, which is a target for corticosteroid-based therapy in EoE patients (36), and aryl hydrocarbon receptor pathway, which has been linked to proton pump inhibitor-mediated inhibition of epithelial proliferation and IL-13 signaling (37), were also identified in EoE-enriched clusters (Figure 6B; Table 1). Furthermore, genes associated with granulocyte macrophage colony-stimulating factor (GM-CSF) were found in EoE-enriched clusters (Figure 6B; Table 1), consistent with GM-CSF signaling playing an active role in EoE epithelial remodeling in mice (38) as well as in eosinophil survival and epithelial crosstalk in EoE patients (39).

**Table 1:** Gene modules predicted to be associated with epithelial populations that are enriched in mice with esophageal squamous cell carcinoma (ESCC) or eosinophilic esophagitis (EoE).

**ESCC-enriched**	
**Module 3**	
Ingenuity Canonical Pathways	-log(*p*-value)
Oxidative Phosphorylation	19.5
Mitochondrial Dysfunction	17.2
Huntington's Disease Signaling	15.1
BAG2 Signaling Pathway	11.4
Sirtuin Signaling Pathway	11.2
**Module 4**	
Ingenuity Canonical Pathways	-log(*p*-value)
Induction of Apoptosis by HIV1	2.79
Osteoarthritis Pathway	2.79
Netrin Signaling	2.53
Proline Biosynthesis I	2.39
Molecular Mechanisms of Cancer	2.39
**Module 5**	
Ingenuity Canonical Pathways	-log(*p*-value)
Oxidative Phosphorylation	9.83
Protein Ubiquitination Pathway	9.34
BAG2 Signaling Pathway	8.16
Sirtuin Signaling Pathway	8.04
FAT10 Signaling Pathway	7.58
**Module 10**	
Ingenuity Canonical Pathways	-log(*p*-value)
EIF2 Signaling	46.6
Regulation of eIF4 and p70S6K Signaling	11.3
mTOR Signaling	10.7
Coronavirus Pathogenesis Pathway	8.61
Mitochondrial Dysfunction	7.37
**EoE-enriched**	
**Module 12**	
Ingenuity Canonical Pathways	-log(*p*-value)
Senescence Pathway	4.9
Glucocorticoid Receptor Signaling	4.74
CSDE1 Signaling Pathway	4.41
Aryl Hydrocarbon Receptor Signaling	4.12
GM-CSF Signaling	3.78

### 3.3. Exposure to EoE inflammation limits esophageal tumorigenesis *in vivo*

**Figure 7:**
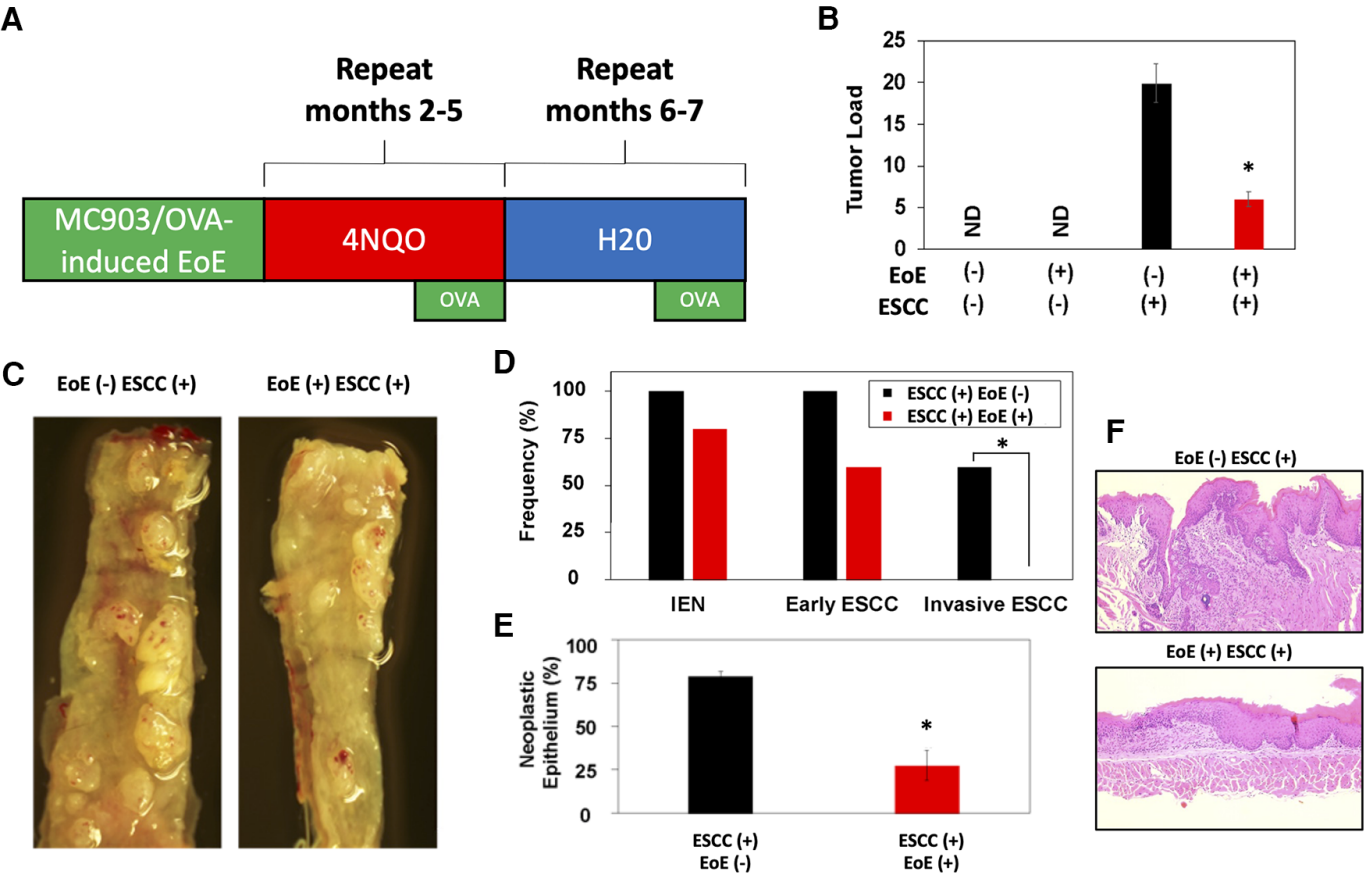
EoE inflammation limits esophageal carcinogenesis *in vivo*. (**A**) Schematic of experimental approach to combined pathology model. C57B6 mice were exposed to MC903/Ovalbumin (OVA) for one month to promote EoE, followed by exposure to the esophageal carcinogen 4-nitroquinoline 1-oxide (4NQO) to induce epithelial tumorigenesis. Esophagi were intermittently challenged with OVA to stimulate EoE-associated inflammation. Mice were either treated with vehicle controls (*n* = 6), MC903/OVA only (*n* = 4), 4NQO only (*n* = 10), or MC903/OVA in combination with 4NQO (*n* = 5). (**B**) Quantification of average tumor load (tumor size in mm x tumor number). ND, not detected. (**C**) Representative esophagi of mice treated with MC903/OVA to promote EoE and/or 4NQO to induce carcinogenesis. (**D,E**) Histological assessment was performed to quantify frequency of esophageal lesion types in (**D**) and percent of esophageal epithelium occupied by neoplastic lesions (squamous dysplasia or greater) with representative images shown in (**E**). *, *p* < 0.05 as calculated by unpaired student's t-test in (**B**) and (**E**) or Fisher's exact test in (**D**).

As our data indicate that EoE and ESCC drive distinct cell fate trajectories in esophageal epithelium and epidemiological data suggest that EoE patients fail to develop esophageal malignancy, we finally paired murine models of EoE and ESCC to explore the functional relationship between the two conditions (Figure 7A). As expected, no tumors were detected in the absence of ESCC-inducing carcinogen treatment either with [ESCC (-) EoE (+)] or without EoE [ESCC (-) EoE (-)] (Figure 7B). In mice treated with ESCC-inducing carcinogen, tumors were detected in 100% of mice in the absence of EoE [ESCC (+) EoE (-)] and 80% of mice in the presence of EoE [ESCC (+) EoE (+)]. Although this difference was not statistically significant, we did detect a significant decrease in tumor load in mice treated with ESCC-inducing carcinogen in the presence of EoE [ESCC (+) EoE (+)] (Figures 7B,C). Additionally, the spectrum of ESCC lesions was shifted in ESCC (+) EoE (+) mice in which no invasive ESCC was detected (Figure 7D). Furthermore, the total percentage of esophageal epithelium occupied by neoplastic lesions was significantly diminished in ESCC (+) EoE (+) mice as compared to their ESCC (+) EoE (-) counterparts (Figures 7E,F).

## 4. Discussion

**Figure 8:**
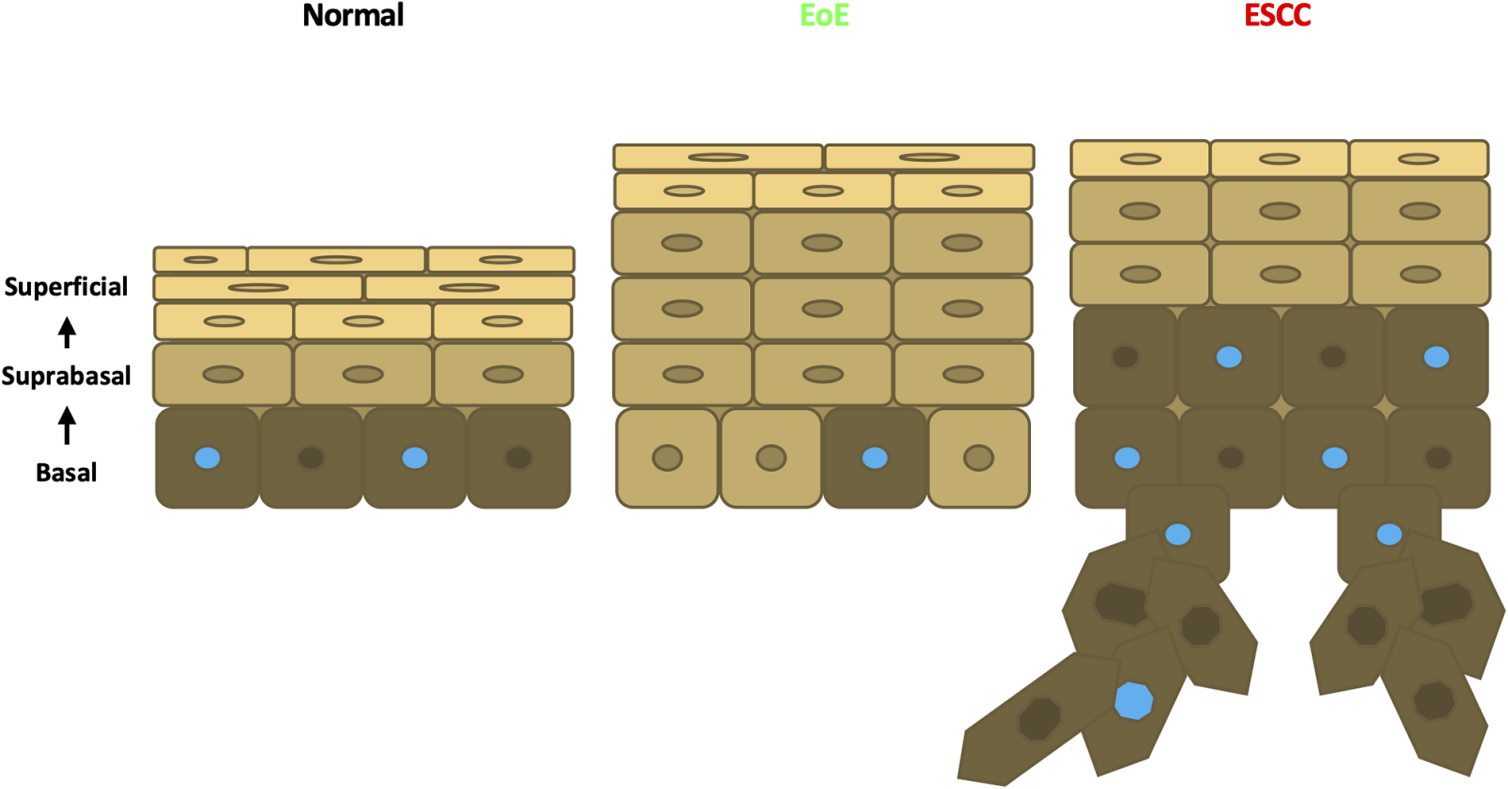
Schematic model of esophageal epithelium in normal, EoE, and ESCC conditions. In normal esophageal epithelium, basal cells give rise to overlying suprabasal cells then terminally differentiated superficial cells. Proliferative cells are denoted with a blue nucleus. In EoE, an expansion of suprabasal cells is present in the epithelium. In ESCC, an expansion of both suprabasal and basal cells is present in the epithelium and encroaches into the underlying stroma.

Esophageal epithelial remodeling has been histologically documented during carcinogenesis and in response to EoE inflammation. Here, we employed scRNA-Seq to compare the impact of these two conditions upon the esophageal epithelial landscape. In mice with EoE, we detect unique accumulation of 4 suprabasal populations and depletion of 2 basal cell populations. These findings are consistent with studies demonstrating impaired squamous differentiation in human subjects with EoE (40). However, it must be noted that while EoE patients often feature basal cell hyperplasia, a histological finding in which basal cells occupy >25% of esophageal epithelial cell height (41), our findings in EoE demonstrate a depletion of basal populations. Here, we have defined basal, suprabasal, and superficial cells using a combination of unbiased bioinformatics-based clustering along with mapping of putative markers of basal and superficial cells onto the identified cell clusters. Although basal cell hyperplasia is assumed to occur *via* expansion of the basal cell compartment, it is possible that basal cell hyperplasia as seen in EoE may instead result from an accumulation of suprabasal cells that fail to differentiate (Figure 8). In an independent scRNA-Seq dataset, we recently validated ATP1B3 as a marker of suprabasal cells in murine esophageal epithelium (35). As such, it will be of interest to determine how EoE inflammation impacts the expression of ATP1B3. In the current study, staining for the cell cycle inhibitor p21 indicates that cells physically located in the basal cell layer of mice with EoE inflammation aberrantly display positivity for p21, which is confined to suprabasal cells in normal esophageal epithelium. Thus, it is further possible that cells located in the basal cell layer of mice with EoE take on features of the suprabasal cell compartment (Figure 8), including exit from the cell cycle. Consistent with EoE-associated alterations in cell cycle within esophageal keratinocytes, a recent human scRNA-Seq study identified an increase in proliferating suprabasal cells, but not proliferating basal cells, in the epithelium of active EoE subjects (42). Identification of proliferating cells in the noted human EoE dataset was based on expression of the proliferation marker *TOP2A* (42). Interestingly, *Top2a* expression is highly upregulated in the EoE-enriched murine population suprabasal 5 in our dataset (Figure 1D) which further displays enrichment of genes associated with G2/M phase of the cell cycle (Figure 4C).

In contrast to our findings in EoE mice, scRNA-Seq in mice with ESCC revealed accumulation of 1 suprabasal population and 2 basal populations, including population basal 1 in which S-phase genes are enriched. Pathway analysis associated cell proliferation pathways, including EIF2 signaling, regulation of eIF4, p70S6 Kinase, and mTOR signaling, with ESCC-enriched cell populations. p21 staining was evident in hyperplastic lesions in esophageal epithelium of mice with ESCC, consistent with oncogene-induced senescence. In mice with ESCC, however, p21 staining remained localized to the suprabasal compartment as was found in normal esophageal epithelium. In sum, these studies suggest that basal cell hyperplasia may be more complex than a mere expansion of basal cells. In EoE, basal cell hyperplasia may reflect an accumulation of suprabasal-like cells with diminished cell cycle activity within the basal layer (Figure 8). It is further possible that EoE-mediated suppression of ESCC-induced tumorigenesis occurs as a direct result of this suprabasal-like cell expansion which limits the presence of a proliferative basal cell pool that drives carcinogenesis (Figure 8). Studies in which molecular markers of individual epithelial cell populations identified using scRNA-Seq are mapped onto esophageal epithelial cells *in situ* will provide valuable insight into basal cell hyperplasia in the context of both EoE and ESCC. Furthermore, identification of strategies to deplete EoE-associated suprabasal populations will be necessary to determine whether these populations play a functional role in EoE-mediated suppression of esophageal carcinogenesis. Additional strategies that examine this functional relationship may target other EoE-associated pathways identified in this study. The aryl hydrocarbon receptor has been implicated as an important regulatory pathway in multiple cancers including ESCC (43, 44). Budesonide, a drug used in the treatment of EoE, targets the glucocorticoid signaling pathway and is an emerging therapeutic agent in cancer (45, 46, 47). The GM-CSF pathway has been demonstrated to facilitate immunotherapeutic treatment of ESCC (48). Finally, the CSDE1 pathway is an inhibitor of stem cell differentiation and is dysregulated in multiple cancers (49, 50). These targets may provide critical insight into the immune and epithelial relationship between EoE and carcinogenesis in the esophagus.

Our scRNA-Seq-based findings in mice with EoE or ESCC coupled with decreased tumor burden in mice with ESCC and EoE as compared to those with ESCC only, supports the premise that exposure to EoE inflammation may alter esophageal epithelial biology in a manner that effectively limits esophageal carcinogenesis. By pairing murine models of EoE and ESCC, we provide the first functional assessment of the relationship between these two disease entities and report that exposure to EoE inflammation limits esophageal carcinogenesis. These findings agree with epidemiological studies in EoE patients that have failed to identify esophageal malignancy in EoE patients (13, 14, 51). As the relationship between allergy and cancer remains elusive, pairing of MC903/OVA-mediated EoE and 4NQO-mediated ESCC provides a novel model in which to functionally dissect the mechanisms through which allergic inflammation impacts cancer initiation and progression. While a number of epidemiological studies report a negative association between allergic inflammation and cancer (14, 15, 16, 17, 18, 19, 20, 21, 22, 51), others report positive or null associations (52, 53). A major limitation of such population-based studies is the inherent heterogeneity of human cohorts with regard to genetics and environmental exposures. A strength of the current study is the use of murine models of esophageal food allergy and cancer that allow for the study of the interaction of these conditions while minimizing effects of such confounding variables. While we anticipate that the methods used herein have great potential to provide mechanistic insight into the relationship between allergy and cancer that may be leveraged for cancer prevention and/or therapy, we must acknowledge the limitations of these methods. It is possible that the protective effects of EoE with regard to esophageal cancer are limited to the MC903/OVA EoE model. Although this model recapitulates features of EoE as found in human patients, including esophageal eosinophilia, basal cell hyperplasia, and food impactions, mice with MC903/OVA-induced EoE display limited intraepithelial eosinophil infiltration as well as the presence of eosinophils beyond the esophagus (29). Various murine models of EoE have been developed (54, 55, 56, 57) and it will be important to determine if the negative impact of EoE inflammation on carcinogenesis is maintained when pairing these models with 4NQO as well as genetically engineered ESCC models (58). Furthermore, it will be of interest to investigate how EoE inflammation influences EAC carcinogenesis. An additional limitation of the current study is a lack of scRNA-Seq data in mice with EoE and ESCC in combination. Such investigations are currently underway and will examine the mutational burden and epigenetic landscape in mice with EoE and ESCC alone and in combination to determine if exposure to EoE inflammation may limit activation of oncogenes and/or enhance activation of tumor suppressors. It will also be of paramount importance to validate findings related to the relationship of EoE and esophageal cancer in tissues from human subjects.

Although the current study examined effects of EoE and ESCC on esophageal epithelium, it is likely that immune-mediated pathways contribute to EoE-mediated suppression of esophageal carcinogenesis. As EoE is clinically characterized by the presence of eosinophils, it is tempting to speculate that these cells may be integral to EoE-mediated suppression of esophageal carcinogenesis. In ESCC, high eosinophil counts are associated with favorable patient outcomes (30, 31, 59, 60). Melanoma patients with high eosinophil counts have also been demonstrated to have prolonged survival following immunotherapy (61). In preclinical models, IL-33 delays metastatic progression of peritoneal cancer *via* induction of an allergic tumor microenvironment in which eosinophils, CD4+ T cells, and macrophages contribute to anti-tumoral effects (62). Moreover, while direct eosinophil-mediated cytotoxicity has been demonstrated *in vitro*, eosinophil-dependent inhibition of tumor initiation *in vivo* occurs by IL-33-mediated effects upon CD8+ T cells in the tumor microenvironment (63). Eosinophil recruitment of CD8+ T cells has also been shown to facilitate tumor rejection *in vivo* (64). Mast cells have also been implicated in EoE pathogenesis (65, 66, 67); however, the relationship of these cell types to cancer remains elusive. The presence of mast cell is associated with improved prognosis in colorectal and breast cancers (68, 69); however, mast cells have also been linked to tumor promotion *via* induction of resistance to anti-PD-1 therapy in melanoma and IL-33-elicited macrophage mobilization in murine models of gastric cancer (70, 71). In ESCC, mast cells have been implicated in tumor progression *via* angiogenesis (72). Here, we utilized epithelium-enriched esophageal tissue fractions for scRNA-Seq which precluded evaluation of the impact of EoE and ESCC on inflammatory cells. In our ongoing studies of mice with EoE and ESCC both alone and in combination, whole esophagi will be subjected to scRNA-Seq to identify immune cells that may contribute to EoE-mediated suppression of esophageal carcinogenesis.

In sum, this investigation unveils marked differences in esophageal epithelial cell remodeling occurring in EoE as compared to ESCC. While esophageal epithelium of mice with ESCC features accumulation of both suprabasal and basal cells, including those with proliferative capacity, esophageal epithelium of mice with EoE features accumulation of suprabasal cells that are largely in G0/G1-phase of the cell cycle and express a gene profile that is associated with senescence. *In vivo* studies combining murine models of EoE and ESCC further demonstrate that exposure to EoE inflammation limits ESCC carcinogenesis. Taken together, our findings raise the possibility that exposure to allergic inflammation may inhibit carcinoma development in the esophagus by pushing esophageal epithelial cells toward a state of stalled differentiation in which they lack the proliferative potential that is needed for carcinogenesis. Should this notion be validated, it may illuminate novel approaches based on epithelial cell fate reprogramming for cancer prevention and/or therapy.

## Data Availability

The datasets presented in this study can be found in online repositories. The names of the repository/repositories and accession number(s) can be found below: https://www.ncbi.nlm.nih.gov/geo/, GSE218118 - Eosinophilic esophagitis-associated epithelial remodeling may limit esophageal carcinogenesis.

## Supplementary material

Supplementary Figure S1

Changes in esophageal epithelial cell populations between untreated, eosinophilic esophagitis (EoE)-treated, and esophageal squamous cell carcinoma (ESCC)-treated esophagi. Uniform Manifold Approximation and Projection plot (UMAP) showing cell populations identified across entire single cell RNA-Sequencing dataset separated by experimental condition.
