# Supplementary material for: Eosinophilic esophagitis-associated epithelial remodeling may limit esophageal carcinogenesis
Source: Front Allergy. 2023 Mar 29;4:1086032. doi: 10.3389/falgy.2023.1086032 (PMC10090679; doi:10.3389/falgy.2023.1086032)
Supplement: Supplementary file 1 [file Image1.pdf]

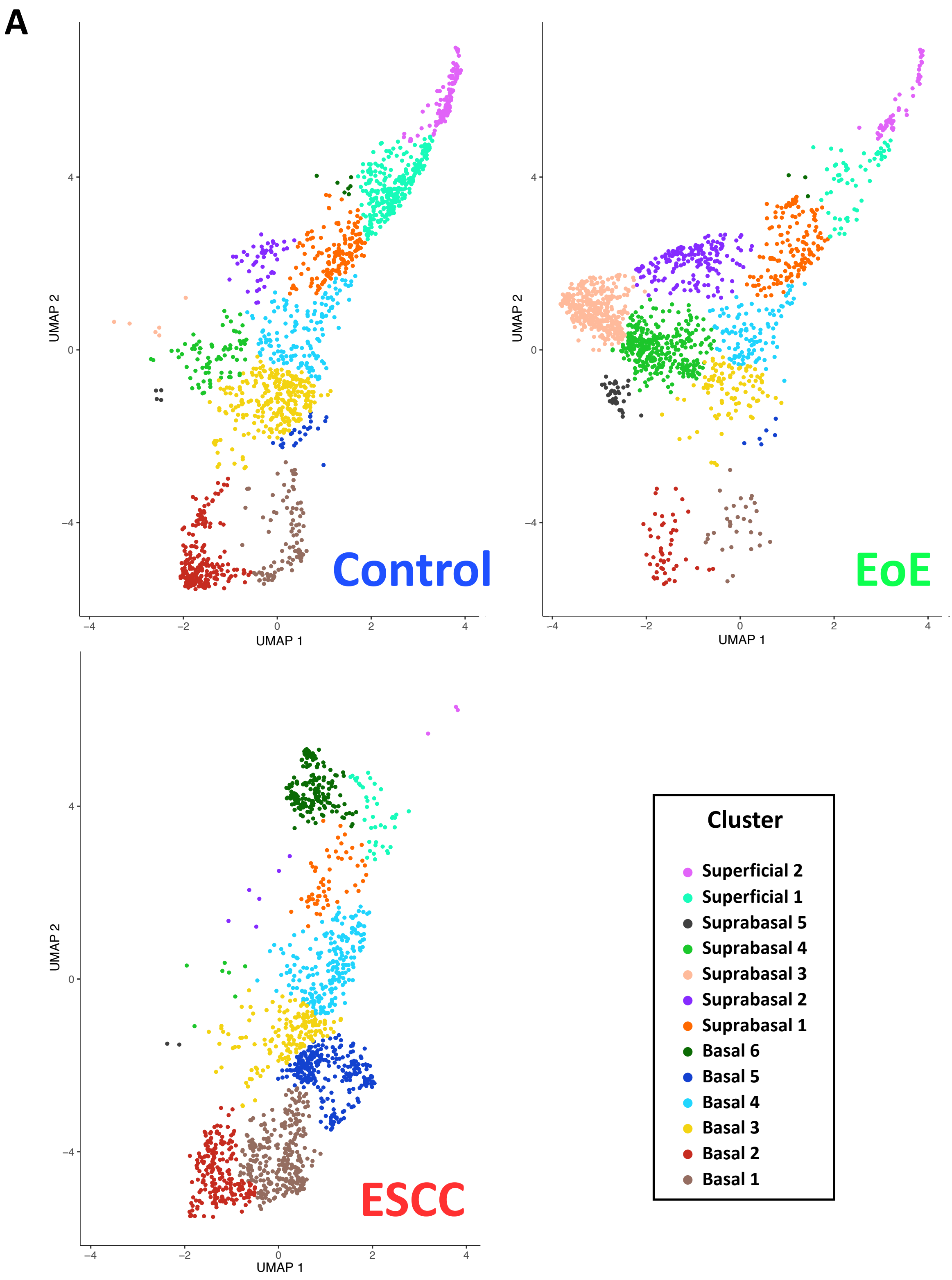

Supplementary Figure S1. Representation of esophageal epithelial cell populations in mice with eosinophilic esophagitis (EoE), esophageal squamous cell carcinoma (ESCC), or untreated controls. Uniform Manifold Approximation and Projection plot (UMAP) showing cell populations identified across entire single cell RNA-Sequencing dataset separated by experimental condition.
